# Extracellular miR-146a-5p Induces Cardiac Innate Immune Response and Cardiomyocyte Dysfunction

**DOI:** 10.4049/immunohorizons.2000075

**Published:** 2020-09-21

**Authors:** Briana K. Shimada, Yang Yang, Jing Zhu, Sheng Wang, Andrew Suen, Stephanie M. Kronstadt, Anjana Jeyaram, Steven M. Jay, Lin Zou, Wei Chao

**Affiliations:** * Translational Research Program, Department of Anesthesiology and Center for Shock, Trauma and Anesthesiology Research, University of Maryland School of Medicine, Baltimore, MD 21201;; † Fischell Department of Bioengineering, University of Maryland, College Park, MD 20740

## Abstract

Previous studies have demonstrated that transient myocardial ischemia leads to release of cellular nucleic acids such as RNA. Extracellular RNA reportedly plays a pivotal role in myocardial inflammation and ischemic injury in animals. RNA profiling has identified that numerous microRNA (miRNAs), such as ss-miR-146a-5p, are upregulated in plasma following myocardial ischemia, and certain uridine-rich miRNAs exhibit strong proinflammatory effects in immune cells via ssRNA-sensing mechanism. However, the effect of extracellular miRNAs on myocardial inflammation and cardiac cell function remains unknown. In this study, we treated adult mouse cardiomyocytes with miR-146a-5p loaded in extracellular vesicles and observed a dose- and TLR7-dependent production of CXCL-2, IL-6, and TNF-α. In vivo, a single dose of myocardial injection of miR-146a-5p induced both cytokine expression (CXCL2, IL-6, and TNF-α) and innate immune cell activation (CD45^+^ leukocytes, Ly6C^mid+^ monocytes, Ly6G^+^ neutrophils), which was significantly attenuated in the hearts of TLR7 KO mice. We discovered that conditioned media from miR-146a-treated macrophages stimulated proinflammatory cytokine production in adult cardiomyocytes and significantly inhibited their sarcomere shortening. Finally, using an electric cell impedance-sensing assay, we found that the conditioned media from miR-146a-treated cardiac fibroblasts or cardiomyocytes impaired the barrier function of coronary artery endothelial cells. Taken together, these data demonstrate that extracellular miR-146a-5p activates multiple cardiac cells and induces myocardial inflammation and cardiomyocyte dysfunction via intercellular interaction and innate immune TLR7 nucleic acid sensing.

## INTRODUCTION

Inflammation is a key component of myocardial ischemic injury (1). Studies have shown that nucleic acids such as cellular RNAs, including microRNAs (miRNAs), are released from initial tissue injury within the myocardium (2–4) and may contribute to the increased myocardial inflammation following ischemia-reperfusion (I/R) injury (4, 5). Systemic administration of nucleic acid binding nanoprobe (5) or RNase (4, 6) leads to reduced myocardial inflammation and infarction, likely by reducing extracellular (ex) RNA levels. Moreover, several of these ex-miRNAs (e.g., ss-miR-146a-5p and miR-133a) induce potent innate immune responses, including cytokine and complement factor production in cultured cells and acute peritonitis after local injection (7, 8). However, it remains unknown how ex-miRNAs such as miR-146a-5p interact with various cardiac cells and whether they induce myocardial inflammation and cardiomyocyte (CM) dysfunction.

miRNAs are small and ss noncoding RNAs that bind to the 3′ untranslated region of a target gene and induce degradation of mRNA or inhibition of translation. miR-146a is among one of the well-studied miRNAs that reportedly regulate innate immune function. When acting in its conventional role within the cell, miR-146a is known as an innate immune regulator, downregulating TRAF-6 and IRAK-1 expression, two key molecules involved in TLR 4 signaling, and attenuating host proinflammatory response to endotoxin (9, 10). In contrast, mature ss-miR-146a-5p, when added to cell cultures or administered in vivo, exhibits a remarkable proinflammatory effect such as cytokine and complement production and immune cell activation (7, 8).

TLRs are a group of innate immune receptors that recognize conserved pathogen-associated molecular patterns presented in different types of pathogens such as bacteria, fungi, and viruses (11). They are expressed either on the surface of cell plasma membrane or intracellular endosomes. TLRs located in endosomes recognize microbial and endogenous nucleic acids such as DNA and RNA (12, 13). TLR 7 in particular is originally known for sensing ssRNA of viral origins such as influenza A and HIV (14, 15), but it can also sense certain miRNAs (7) and other small RNA (15, 16).

In this study, we tested the hypothesis that exogenous ss-miR-146a-5p is an innate immune mediator capable of inducing myocardial inflammation and CM contractile dysfunction. We showed that mouse CMs produced proinflammatory cytokines in response to ex vesicles (EVs) loaded with miR-146a-5p. We demonstrated that intracardiac injection of miR-146a-5p induced a robust cardiac innate immune response via a TLR7-dependent mechanism. Moreover, we identified that miR-146a-5p activated heterocellular cross-talk signaling among various cardiac cell types in vitro and led to impairment of coronary artery endothelial cell barrier function and CM contractile function. These findings provide new insight into the effect of ex-miRNAs on innate immune response and function of cardiac cells.

## MATERIALS AND METHODS

### Materials

miR-146a-5p and miR-146a-5p_U → A_ mutant were synthesized by Integrated DNA Technologies (Coralville, IA). Imiquimod (R837) was purchased from InvivoGen (San Diego, CA). Lipofectamine 3000 was from Thermo Fisher Scientific (Waltham, MA). LPS, collagenases B and D, gelatin, and pancreatin were from Sigma-Aldrich (St. Louis, MO). Laminin was from Roche Diagnostics (Basel, Switzerland). Collagenase 2 was acquired from Worthington Biochemical, (Lakewood Township, NJ). Phthalo blue was acquired from Liquitek (Cincinnati, OH).

### Animals

Wild-type (WT; C57BL/6) and TLR7 knockout (TLR7 KO; TLR7^tm1Flv^/J, stock no. 008380) mice were purchased from the Jackson Laboratory (Bar Harbor, ME). Adult male mice were between 9 and 12 wk of age, weighed between 20 and 30 g, and were gender and age matched. Neonatal rats (Sprague Dawley) were purchased from Charles River (Wilmington, MA). Mice were housed and fed as specified in a previous publication (17). WT mice were housed for at least 1 wk prior to being used in experiments. The animal protocols were approved by the Institutional Animal Care and Use Committee, University of Maryland School of Medicine (Baltimore, MD). All animal experiments were performed in compliance with the guidelines of the National Institutes of Health (Bethesda, MD). A simple randomization method was used to assign animals to various experimental conditions. All strains and reagents were blinded to the operator(s) for the in vivo studies unless otherwise stated.

### EV isolation, preparation, and miRNA loading

EVs were isolated, prepared, and loaded as described previously (18). Briefly, EVs were isolated from HEK293T cells by differential ultracentrifugation, washed, and filtered using a 0.2-μm syringe filter. EVs were quantified for size and concentration using a NanoSight LM10. Following isolation, EVs were sonoporated in a water bath sonicator (VWR Symphony) at 35kHz. Nucleic acid loading was quantified using Quant-iT PicoGreen Assay Kits (Life Technologies) following extensive washing using a 300-kDa MWCO filter to remove excess unincorporated RNAs.

### Intramyocardial miRNA injection

Mice were anesthetized by i.p. injection of ketamine (120 mg/kg) and xylazine (4 mg/kg). After confirming adequate general anesthesia by toe pinch, mice were laid down in a supine position on a dedicated heating pad to maintain the core body temperature of the mice at 37°C, fixed to the plate, and intubated. Three hundred microliters of sterile saline was given s.c. prior to the surgery. To reach the anterior wall of the heart, an incision was made in the left midclavicular line to expose the underlying muscles. The pectoralis major muscle and underlying pectoralis minor muscle was bluntly separated to expose the ribs. The fifth intercostal space was pierced and enlarged with a retractor opening the thorax. The pericardium was stripped exposing the heart anterior wall. Sixty microliters of a freshly prepared mix of miRNA and lipofectamine 3000 (ratio 1.6:1 in volume) was injected into the left ventricular anterior wall using a 1-ml BD insulin syringe with a 31-gauge needle (19). To reduce pain at the injection site, 0.1 mg/kg buprenorphine was injected s.c. Mice were kept on a ventilator until they established spontaneous breathing, responded to toe pinch, and displayed the righting reflex. They were then extubated and moved to a mouse postanesthesia care unit container on a warm pad with supplemental O_2_ for 1–2 h before transfer to a clean cage.

### RNA extraction and quantitation

RNA from heart tissue was extracted using TRIzol (Sigma-Aldrich) and quantified using the Nanodrop as reported previously (4).

### Isolation of adult mouse ventricular myocytes for IonOptix

WT and TLR7 KO mice were heparinized (1000 IU/kg, i.p.) and anesthetized (ketamine/xylazine, i.p.). The heart was then quickly removed from the chest and retrograde perfused through the aorta at a constant flow of 3.0 ml/min at 37°C with a Ca^2+^-free HEPES-based buffer containing 130 mM NaCl, 5.4 mM KCl, 0.5 mM MgCl_2_, 0.33 mM NaH_2_PO_4,_ 22 mM glucose, and 25 mM HEPES (pH 7.4) for 2–3 min. Following perfusion with the Ca^2+^-free buffer, enzymatic digestion was initiated by perfusion with the Ca^2+^-free buffer containing 0.4 mg/ml of collagenase B, 0.3 mg/ml of collagenase D, and 0.3 mM CaCl_2_ for 15–20 min until the heart became soft to the touch. The heart was then transferred to a 60-mmculturedish containing thesameCa^2+^ buffer and collagenases with 2 mg/ml BSA and 0.7 mM CaCl_2_. The digested heart tissue was then filtered with a sterile, 100-μm nylon mesh filter into a 15-ml conical tube. CMs settled by gravity for 10 min before they were transferred to another 15-ml conical tube containing Ca^2+^-free buffer with 1.2 mM CaCl_2_ and 2mg/ml BSA and allowed to settle for another 10 min. CMs were then transferred to Ca^2+^ Tyrode buffer containing 140 mM NaCl, 5.4 mM KCl, 1.8 mM CaCl_2_, 0.5 mM MgCl_2_, 0.33 mM NaH_2_PO_4_, 11 mM glucose, and 5 mM HEPES (pH 7.4) for IonOptix experiments (20).

### IonOptix

A glass coverslip was placed in a chamber mounted on the stage of an inverted microscope (Nikon Eclipse, Tokyo, Japan). CMs were loaded on to the coverslip, allowed to settle by gravity and perfused at 1 ml/min at 37°C with Ca^2+^ Tyrode Buffer. The cells were field stimulated at 2 Hz using a MyoPacer (IonOptix, Westwood, MA). A video-based edge detector was used to capture and convert changes in cell length during shortening and relengthening into an analogue voltage signal. Contractility was assessed using percent sarcomere length shortening as previously described with minor modifications mentioned above (21).

### Flow cytometry

After digestion of the hearts using the method for CM isolation, noncardiomyocyte cells were filtered through 100- and 40-mm cell strainers (VWR, Radnor, PA) and centrifuged at 134 × *g* for 10 min. Cells were resuspended in 400 μl of PBS and 100 μl was taken and incubated with the following Abs at the various dilutions listed: 1:2000 CD45 PE (catalog no. 553081; BD Biosciences), 1:100 Ly6G BV421 (catalog no. 562737; BD Biosciences), 1:1000 Ly6C PECy7 (catalog no. 25-5932-80; eBioscience), 1:100 F4/80 Alexa Fluor 647 (catalog no. 565853; BD Biosciences), and 1:1000 Zombie NIR (catalog no. 423105; BioLegend). The samples were then fixed with 1% paraformaldehyde and run on a BD LSR II.

### Adult mouse CM culture

Ninety-six–well plates were coated with 10 μg/ml laminin for at least 2–4 h prior to CM culture. WT and TLR7 KO mice were anesthetized and the heart was quickly removed from the chest and retrograde perfused at a constant flow of 3.0 ml/min at 37°C for 2–3 min with a Ca^2+^-free bicarbonate based buffer containing 120 mM NaCl, 5.4 mM KCl, 1.2 mM MgSO_4_, 1.2 NaH_2_PO_4_, 5.6 mM glucose, 20 mM NaHCO_3_, 10 mM 2,3-butanedione monoxime (Sigma-Aldrich), and 5 mM taurine (Sigma-Aldrich), gassed with 95% O_2_-5% CO_2_. Following perfusion with the Ca^2+^-free buffer, enzymatic digestion was initiated by perfusing with a collagenase buffer containing 0.4 mg/ml collagenase type B, 0.3 mg/ml collagenase type D, and 0.03 mg/ml protease type XIV (Sigma-Aldrich). All solutions were filtered with a 0.22-μm filter. Hearts were perfused with the collagenase buffer for 15–20 min until heart became soft. Collagenase buffer was then washed out by perfusing again with Ca^2+^-free buffer for 2–3 min. Following, CMs were isolated by mechanically pulling them apart and were filtered using a sterile 250-μm filter (22). Myocytes were then allowed to settle by gravity. CMs then underwent calcium upload with increasing concentrations of calcium (0.06, 0.24, 0.6, 1.2 mM) for 10 min each. They were then resuspended in M199 medium supplemented with 5% FBS, 100 U/ml penicillin/streptomycin, and 25 μM blebbistatin (Sigma-Aldrich) and seeded at 10,000 cells per well in 96-well plates. Two hours after seeding, the media was changed to M199 medium supplemented with 0.1 mg/ml BSA, 100 U/ml penicillin/streptomycin, 1× insulin/transferrin/sodium selenite (Sigma-Aldrich), and 25 μM blebbistatin (22).

### Isolation and culture of bone marrow–derived macrophages

Macrophages were isolated and cultured from WT and TLR7 KO mice as described previously (7).

### Rat neonatal CM and fibroblast culture

Rat neonatal CMs were prepared as previously described with minor modifications (7). Briefly, fibroblasts were cultured by plating cells on 10-cm dishes for 70–90 min. When they reached confluency, fibroblasts were passaged onto a 12-well plate at a density of 0.6 × 10^6^ cells per well. Neonatal CMs were plated on either a 96- or 24-well plate, precoated with 5 mg/ml fibronectin (Sigma-Aldrich) and 0.2 mg/ml gelatin (Sigma-Aldrich), and seeded at a density of 0.8 × 10^5^ cells per well (96-well) or 0.4 × 10^6^ cells per well (24-well) and incubated in CO_2_ incubator at 37°C for; ~36 h before experiments.

### Cytokine detection

CXCL2, TNF-α, and IL-6 from culture media were tested with commercially available ELISA kits (R&D Systems, Minneapolis, MN and PeproTech, Rocky Hill, NJ).

### Human coronary artery endothelial cell culture

Human coronary artery endothelial cells (HCAECs) were purchased from Lonza (Morristown, NJ). HCAECs were cultured in endothelial basal medium-2 (Lonza) supplemented with EGM-2MV Single Quotes Medium containing endothelial basal medium-2, 0.5 ml of human epidermal growth factor, 0.5 ml of vascular endothelial growth factor (VEGF), 0.5 ml of R3-insulin-like growth factor-1 (R3-IGF-1), 0.5 ml of ascorbic acid, 0.2 ml of hydrocortisone, 2.0 ml of human fibroblast growth factor (hFGF-β), 0.5 ml of gentamicin/amphotericin-B, and 5% FBS. For experiments, cells at passages three to six were used and the serum was decreased to 2% FBS to prevent cell proliferation. Cells were cultured under standard conditions (37°C, 5% CO_2_) in a Steri-Cycle Incubator (Thermo Fisher Scientific).

### Electric cell substrate impedance–sensing system

HCAECs were passaged and seeded onto an eight-well electric cell substrate impedance–sensing (ECIS) array (8W10E+; Applied BioPhysics) at a cell density of 1.8 × 10^5^ cells/well. In some experiments, HCAECs were directly treated with 50 nmol/l of miR-146a or lipofectamine. In the other experiments, 50% conditioned medium from rat neonatal CMs or fibroblasts was used to treat the HCAECs. Transendothelial resistance of HCAECs were measured continuously for 10 h at frequency of 4000 Hz. Normalized resistance to each well’s own baseline was used to assess the permeability. Treatments were measured in duplicates or triplicates and averaged to plot as a single curve with error bars representing mean ± SD.

### Statistical analysis

Statistical analysis was performed using GraphPad Prism 8 software (GraphPad, La Jolla, CA). Unless stated otherwise, the distributions of the continuous variables were expressed as the mean ± SD. For cytokine levels below the detection limit, the values input at the detection limit were used. D’Agostino and Pearson (single-comparison) or Kruskal–Wallis (multiple-comparison) normality tests were used to evaluate the normality of the data. If normal, unpaired two-tailed *t* test (single-comparison) or one-way or two-way ANOVA with Tukey post hoc test (multiple-comparison) were used. If the data did not pass the normality test, nonparametric tests (Mann-Whitney for single-comparisons or Dunn test for multiple comparisons) were used to calculate the *p* values. The null hypothesis was rejected for *p* < 0.05 with two-tailed test.

## RESULTS

### EVs loaded with miR-146a-5p induce proinflammatory cytokines in adult mouse CMs

**FIGURE 1.**
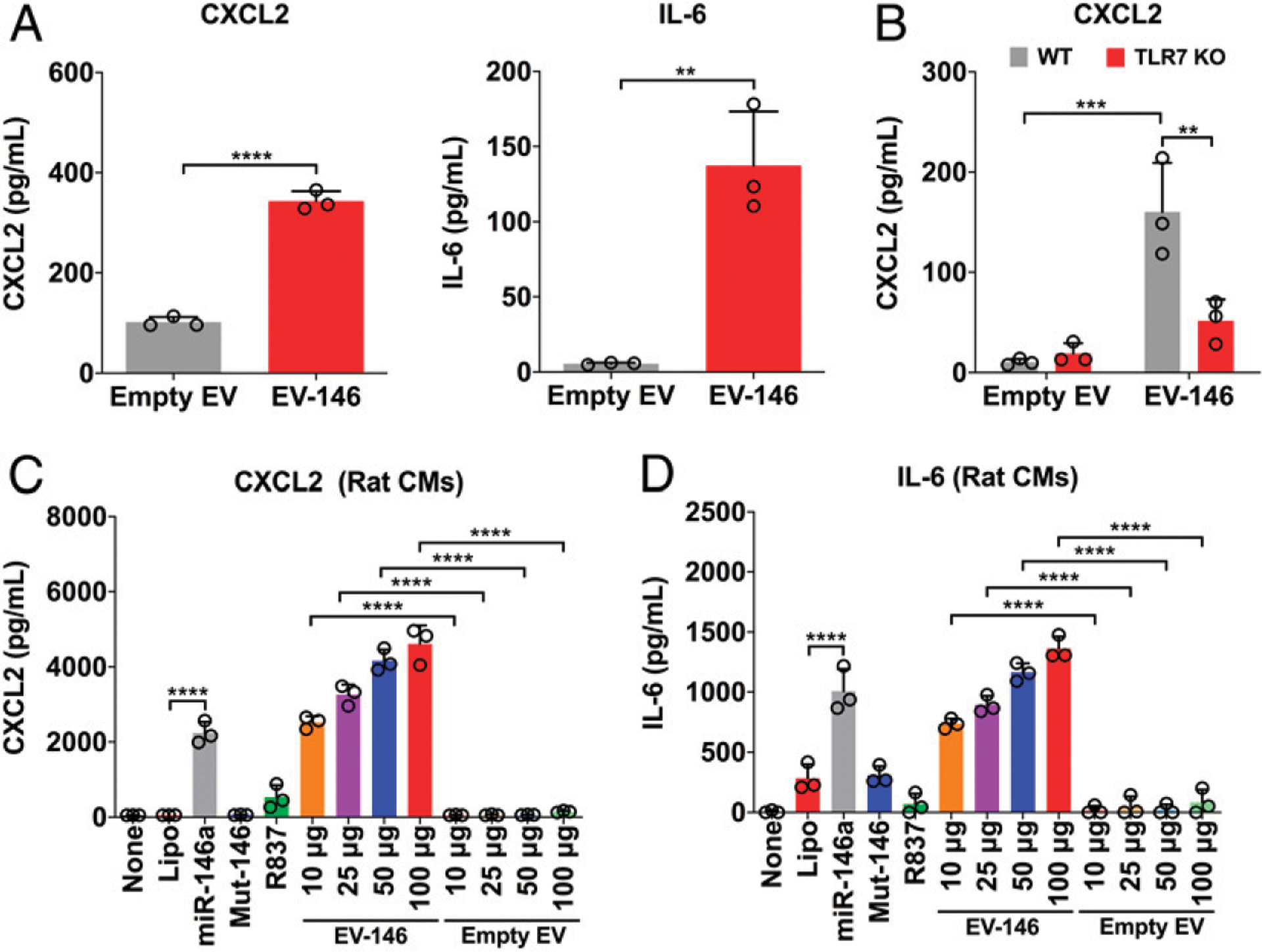
Engineered EVs loaded with miR-146a-5p induce a proinflammatory response in CMs. EVs were isolated from HEK293T cells and loaded with or without miR-146–5p mimics as described in the Materials and Methods. Cells were incubated with prewarmed serum-free RPMI-1640 medium containing 0.05% BSA for 1 h prior to treatment. (**A**) WT adult mouse CMs were treated with 50 mg of empty or miR-146a-5p–loaded EVs. (**B**) WT or TLR7 KO CMs were treated with 50 mg of empty or miR-146a-5p–loaded EVs. (C and D) Neonatal rat CMs were treated with Lipo, miR-146, Mut-miR-146, R837 (TLR7 agonist), and various doses of EVs loaded with miR-146a-5p for 16–18 h. CXCL2 and IL-6 productions were assessed by ELISA. Experiments were performed in triplicates and repeated independently two to three times. Each error bar represents mean ± SD. Data in (A) were analyzed with two-tailed t test. (B) was analyzed with two-way ANOVA with Tukey post hoc test. (C) and (D) were analyzed with one-way ANOVA with Tukey post hoc test. **p < 0.01, ***p < 0.001, ****p < 0.0001. EV-146, EV-miR-146a-5p; Lipo, lipofectamine; miR-146, miR-146a-5p (150 nmol/l); mut-146, miR-146a-5p _U → A_ mutant (150 nmol/l).

**FIGURE 2.**
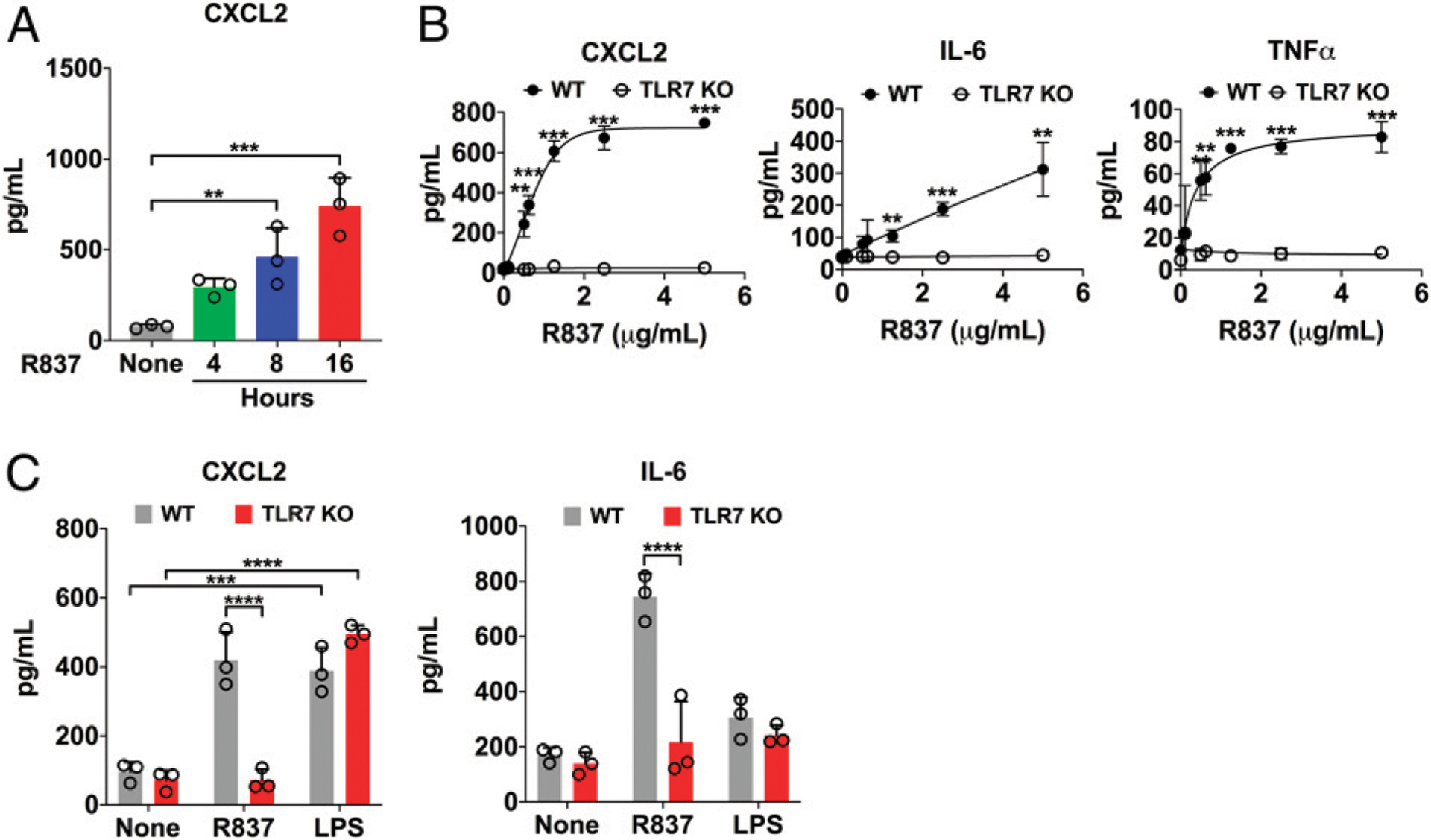
R837 induces inflammatory cytokines in adult mouse CMs via a TLR7-dependent mechanism. Cell culture medium was changed to prewarmed, serum-free M199 medium containing 0.05% BSA for 1 h prior to treatment. CMs were treated with R837 and LPS at the specified doses for 16–18 h before the media was collected. (**A**) Time course of CXCL2 production after treatment with 1.25 μg/ml R837. (**B**) Dose-response curves of R837 in WT and TLR7 KO CMs. (**C**) Cytokine production in WT and TLR7 KO CMs treated with 1.25 μg/ml R837 or 500 ng/ml LPS. Each error bar represents mean ± SD. Time course in (A) was analyzed with one-way ANOVA with Tukey post hoc test. Dose curves in (B) were analyzed with multiple two-tailed *t* tests. Data in (C) were analyzed with two-way ANOVA with Tukey post hoc test. Experiments were performed in triplicates and repeated independently two to three times. **p < 0.01, ***p < 0.001, ****p < 0.0001.

We treated adult mouse CMs in vitro with ss–miR-146a-5p in the presence of lipofectamine and observed no significant CXCL2 response, which was probably due to poor in vitro nucleic acid transfection efficiency in adult mouse CMs as previously reported (23). To circumvent this, we prepared EVs, a natural carrier for circulating miRNAs in vivo, from HEK293 cells as an alternative delivering carrier for miRNA (18) and treated both adult mouse and neonatal rat CMs with the engineered EV-miR-146a-5p. As shown in Fig. 1A, we observed significantly elevated CXCL2 and IL-6 production in adult mouse CMs treated with 50 μg of EVs loaded with miR-146a compared with empty EVs. The EV-miR-146a–induced cytokine response appeared to be mediated via TLR7 as minimal cytokines were produced in CMs lacking TLR7 (Fig. 1B). In rat neonatal CMs (Fig. 1C, 1D), there was a dose-dependent increase in CXCL2 and IL-6 production in cells treated with EVs loaded with miR-146a but not empty EVs (Fig. 1C). Unlike adult mouse CMs, neonatal rat CMs did respond to miR-146a-5p packed in lipofectamine. Moreover, we treated adult mouse CMs with the TLR7 agonist R837 (24) and noticed that R837 induced a time- and dose-dependent increase in CXCL2, IL-6, and TNF-α responses (Fig. 2A, 2B). Similar to EV-miR-146a-5p, this effect appeared to be TLR7-dependent as CMs deficient of TLR7 failed to respond to R837, but responded normally to LPS, a TLR4 agonist (Fig. 2C). Of note, the miRNA doses were chosen for the in vitro studies based on pharmacological consideration. The EC_50_ for miR-146a-5p-induced IL-6 production in cultured macrophages is between 5 and 10 nM (data not shown).

### Intracardiac injection of miR-146a-5p induces inflammation in the heart

**FIGURE 3.**
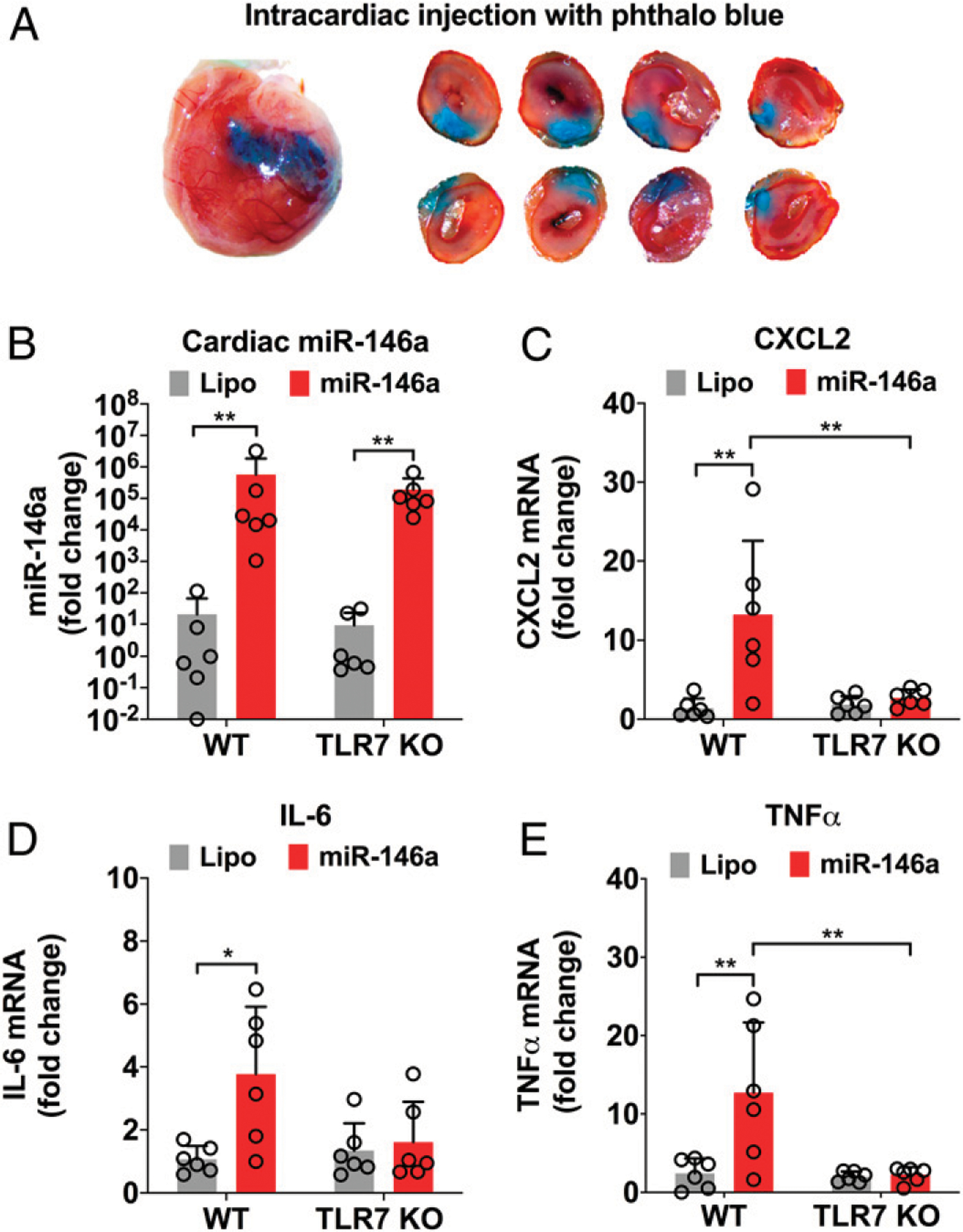
Intracardiac injection of miR-146a-5p results in TLR7-dependent cytokine production in the heart. (**A**) Sixty microliters of phthalo blue dye was injected into the anterior wall of left ventricle to outline the area of injection. Hearts were sectioned into four slices and imaged on both sides to show injection primarily stays in the myocardium. (**B**) miR-146a-5p expression in the heart 24 h after injection of Lipo or miR-146a-5p in WT and TLR7 KO mice. (**C–E**) Quantitative RT-PCR analysis of cytokine genes in the hearts injected with Lipo or miR-146–5p. Each error bar represents mean ± SD (*n* = 6 mice in each group). Data in (B) were analyzed using a Mann-Whitney nonparametric test as the data did not pass the normality test. Data in (C)–(E) were analyzed with two-way ANOVA with Tukey post hoc test. *p < 0.05, **p < 0.01. Lipo, lipofectamine; miR-146, miR-146a-5p.

**FIGURE 4.**
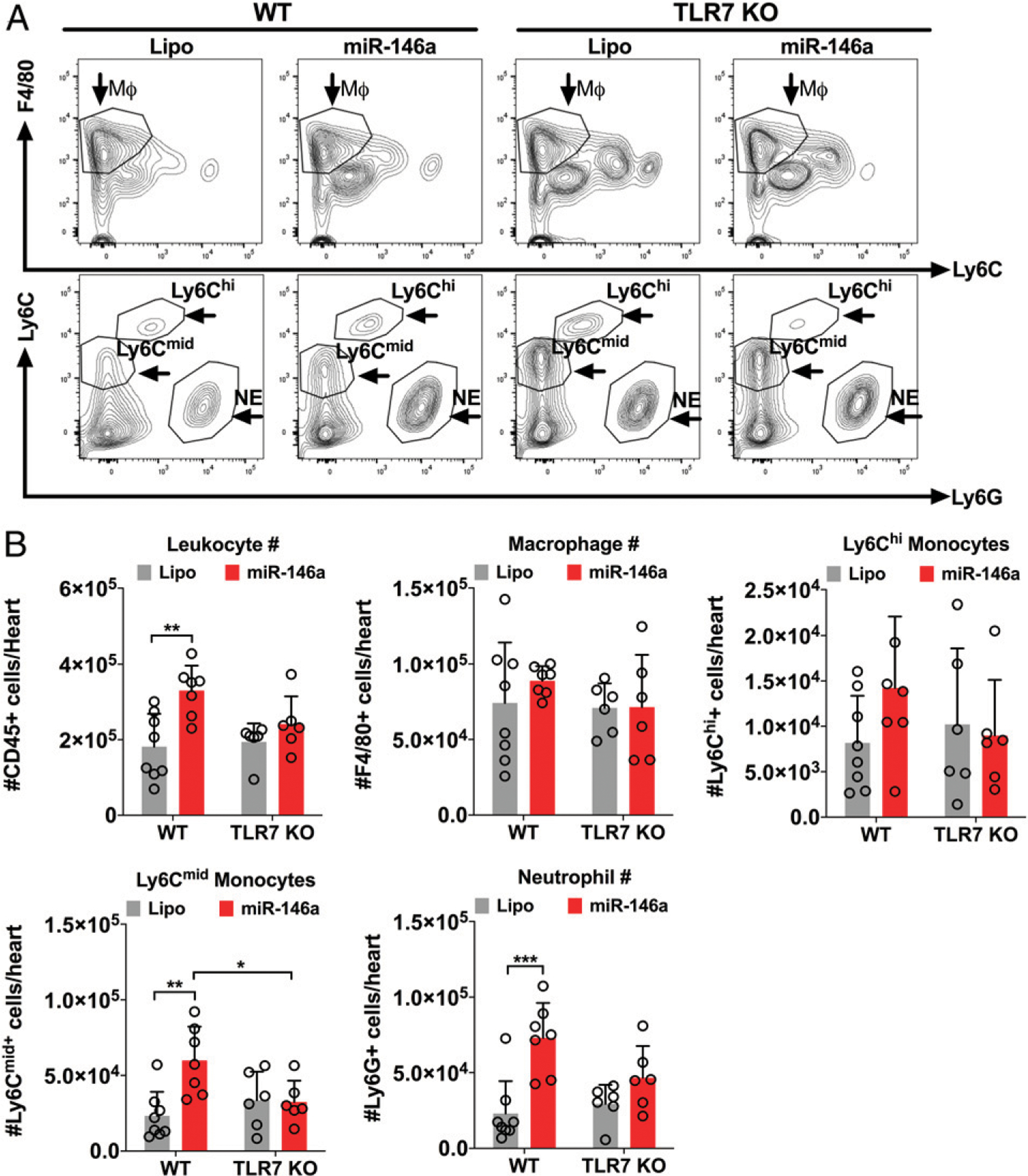
Intracardiac injection of miR-146a-5p induces neutrophil and monocyte migration into the myocardium via a TLR7-dependent mechanism. WT and TLR7 KO male mice were injected with 6 mg of miR-146a-5p mimics or Lipo alone into the myocardium. Twenty-four hours later, the heart was digested using collagenases and filtered. The immune cells were then collected and the total number of macrophages (MΦ), neutrophils (NE), and monocytes (MO) were determined by flow cytometry as detailed in Materials and Methods. (**A**) Representative flow contour plots gated for Ly6G^+^, F4/80^+^, Ly6C^mid^, and Ly6C^hi^ in each group. (**B**) The number of immune cells in each group. Each error bar represents mean ± SD (*n* = 6–8 mice per group). Data in (B) were analyzed using two-way ANOVA with Tukey post hoc test. *p < 0.05, **p < 0.01, ***p < 0.001. Lipo, lipofectamine; miR-146a, miR-146a-5p.

Next, we tested the ability of miRNA-146a-5p to activate myocardial innate immune response in vivo by direct intracardiac injection. We first established and visualized myocardial distribution by injecting 60 μl of a sterile filtered 1% phthalo blue dye mixed with sterile saline into the myocardium (Fig. 3A). We then injected miR-146a-5p (6 μg/heart) or control vehicles into the hearts of WT or TLR7 KO mice. This concentration was chosen based on previous publications using a similar range (between 1 and 10 μg) of miRNAs to inject into the heart (19, 25). Twenty-four hours after injection, myocardial expression of miR-146a-5p was markedly upregulated to a similar degree in the hearts of both WT and TLR7 KO as compared with vehicle alone (Fig. 3B). There was a significant increase in CXCL2, IL-6, and TNF-α transcripts in the hearts of WT mice, but not in those of TLR7 KO mice, following miR-146a injection (Fig. 3C–E). Moreover, we assessed whether myocardial injection of miR-146a-5p promotes immune cell infiltration into the heart and whether this recruitment was also TLR7-dependent. Using flow cytometry (Fig. 4A) with the gating strategy shown on Supplemental Fig. 1, we analyzed the noncardiomyocyte cells isolated from the mouse heart and found that compared with the vehicle-injected hearts, miR-146a-injected hearts had a significantly higher number of total CD45^+^ leukocytes (3.30 ± 0.67 × 10^5^ versus 1.81 ± 0.87 × 10^5^ cells per heart, *p* < 0.01), Ly6C^mid+^ monocytes (5.98 ± 2.25 × 10^4^ versus 2. 32 ± 1.59 × 10^4^ cells per heart, *p* < 0.05), and Ly6G^+^ neutrophils (7. 30 ± 2.31 × 10^4^ versus 2.30 ± 2.14 × 10^4^ cells per heart, *p* < 0.01; Fig. 4B). There was no difference in Ly6C^high^ monocytes between the two groups. In comparison, TLR7 KO hearts injected with the same dose of miR-146a-5p had similar numbers of monocytes and neutrophils as compared with the vehicle-injected hearts. Of note, vehicle injection alone had no impact on cardiac leukocyte numbers compared with saline controls (Supplemental Fig. 2).

### miR-146a-5p stimulates proinflammatory interaction between CMs and noncardiomyocytes

**FIGURE 5.**
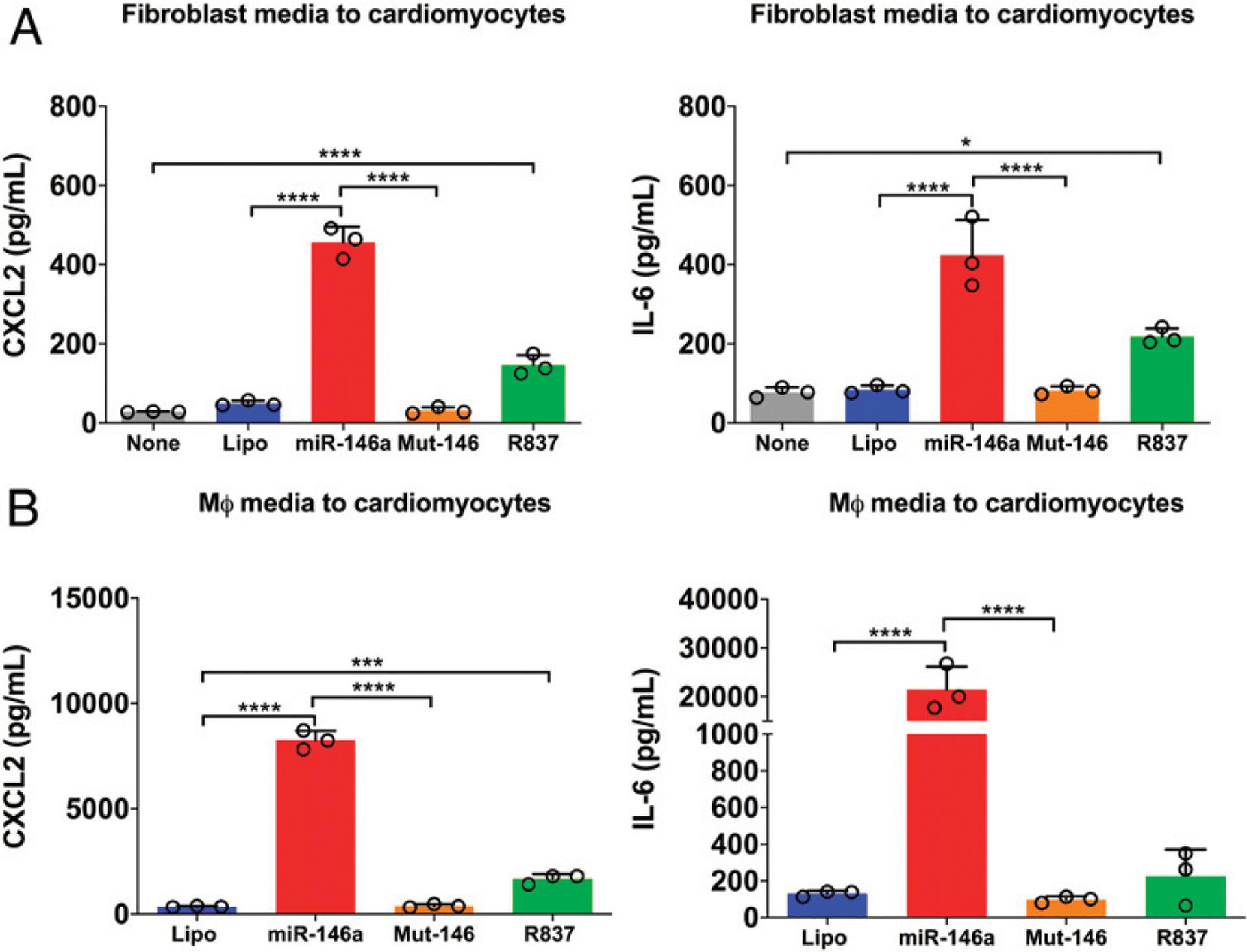
Proinflammatory cross-talk among CMs and noncardiomyocytes. Rat cardiac fibroblasts or mouse macrophages were treated for 16–18 h with the indicated treatments. The conditioned media was collected and used at 50% concentration to treat either adult mouse CMs or neonatal rat CMs, respectively, for 16–18 h. (**A**) CXCL2 and IL-6 production in adult mouse CMs treated with fibroblast-conditioned media. (**B**) CXCL2 and IL-6 production in neonatal rat CMs treated with macrophage-conditioned media. Experiments were performed in triplicates and repeated at least twice. Data in (A) and (B) were analyzed using one-way ANOVA with Tukey post hoc test. Each error bar represents mean ± SD. R837 (1.25 μg/ml). *p < 0.05, ***p < 0.001 ****p < 0.0001. Lipo, lipofectamine; MΦ, macrophage; miR-146a, miR-146a-5p (150 nmol/l); mut-146, miR-146a-5p _U → A_ (150 nmol/l).

The heart possesses multiple cell types: CMs, fibroblasts, residential macrophages, and vascular endothelial cells. These cardiac cells interact with each other under normal and pathological conditions (26, 27). To understand the effect of miR-146–5p on cardiac cell functions, we tested whether miR-146a-5p could result in “cross-talk” between different types of cardiac cells using various cellular functional assays. We first treated rat cardiac fibroblasts with vehicles, miR-146a, the inactivemiR-146a_U → A_ mutant, ortheTLR7 agonist R837. We then collected the culture media 16–18 h later and treated adult mouse CMs with 50% of the conditioned media for 16–18 h. We discovered that mouse adult CMs responded with increased CXCL2 and IL-6 production to the conditioned media from cardiac fibroblasts treated with miR-146a-5p, but not vehicles or the miR-146a-5p mutant (Fig. 5A). Similar results were observed with miR-146a-5p-treated mouse macrophage culture media in rat CMs (Fig. 5B). It is noteworthy that by design, cells of different species (i.e., rat versus mouse) were used in this set of experiments and the cytokines detected were determined to be released from the conditioned medium-treated cells, not from the conditioned media, because the ELISAs used were highly specific with no cross-activity between mouse and rat.

### miR-146a-5p disrupts cardiac endothelial barrier function via cardiac myocytes and fibroblasts

**FIGURE 6.**
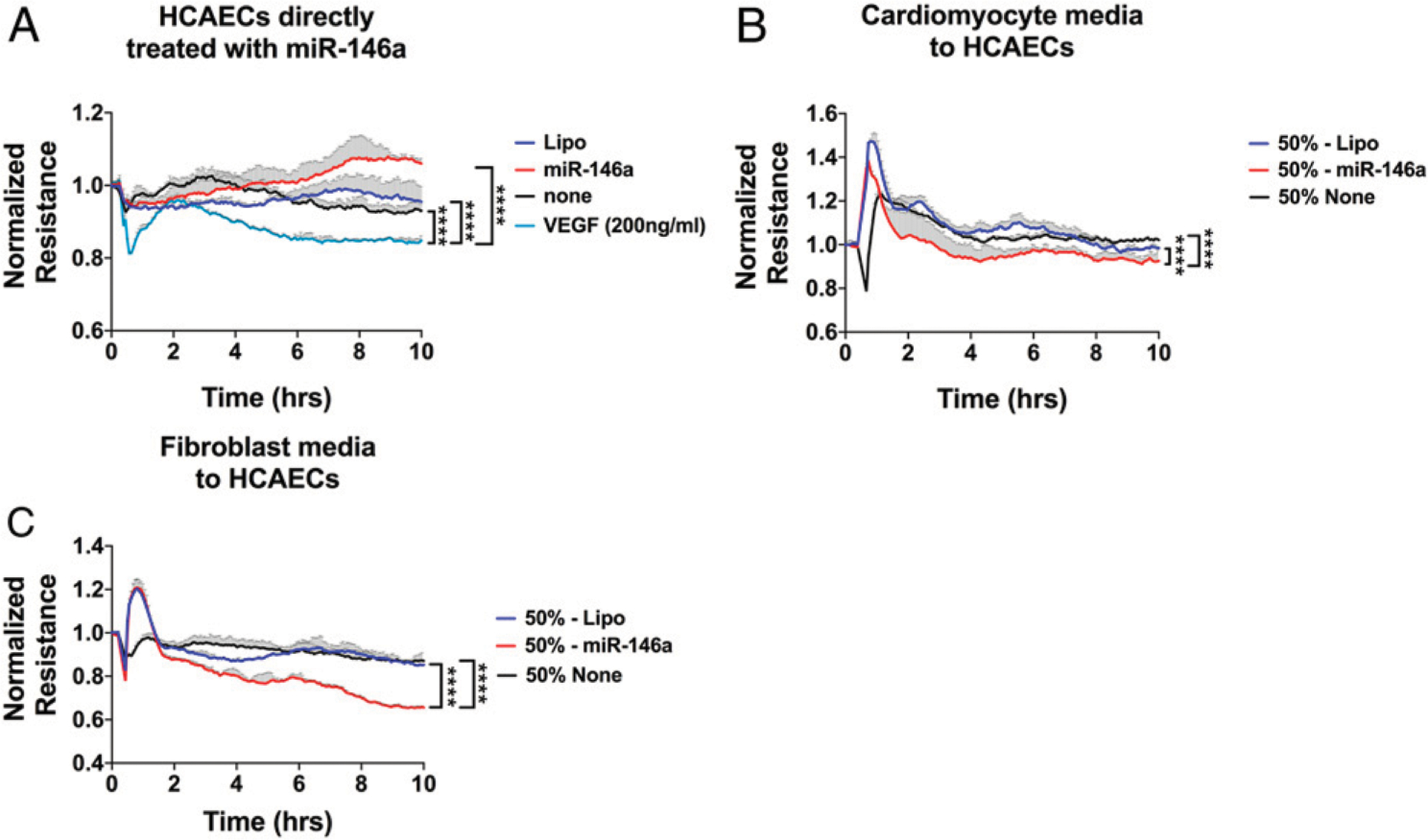
Cardiac fibroblast and CM-conditioned media disrupt the barrier function of coronary artery endothelial cells. Fibroblasts and CMs were treated for 16–18 h with either Lipo or miR-146a (50 nmol/l). Media were collected under sterile conditions, centrifuged, and stored at −80°C. HCAECs were passaged and seeded on eight-well ECIS plates at a density of 1.8 × 10^5^ cells per well. Once confluency was reached, HCAECs were treated for 10 h with miR-146a or Lipo in some experiments (**A**) or 50% of conditioned media in others (**B** and **C**). (A) HCAECs were treated with miR-146a-5p (50 nmol/l) or VEGF (200 ng/ml) for 10 h. (B) HCAECs treated with 50% of conditioned media from CMs. (C) HCAECs treated with 50% of conditioned media from cardiac fibroblasts. Experiments were performed in duplicates and repeated at least twice. Data curves were analyzed using a repeated measures one-way ANOVA with Tukey post hoc test. ****p < 0.0001. Lipo, lipofectamine; miR-146a, miR-146a-5p.

During myocardial inflammation, the endothelial cell barrier is disrupted and gap junctions become impaired (28, 29), which can lead to increased extravascular migration of monocytes and neutrophils into the myocardium. We tested the barrier function of HCAECs using an ECIS system, which quantifies endothelial barrier function by measuring the resistance of electric current applied to an endothelial cell monolayer on top of a gold interdigitating electrode (30). Although VEGF, a well-known barrier disrupting mediator, induced a time-dependent reduction in the electric resistance in the ECIS assay, similar treatment with miR-146a-5p had no impact on the endothelial barrier function (Fig. 6A). This was probably because of the lack of TLR7 expression in endothelial cells (31), which we confirmed using quantitative RT-PCR in these HCAECs (data not shown). Thus, we explored the possibility of whether miR-146a-5p-activated cellular responses in other cardiac cells such as CMs or fibroblasts could indirectly result in endothelial barrier disruption. To test this, we treated rat cardiac myocytes and fibroblasts with miR-146a-5p for 16 h. We collected and used 50% conditioned media to treat the HCAECs for 10 h. As shown in Fig. 6B and 6C, conditioned media from either cardiac myocytes or fibroblasts treated with miR-146a-5p, but not the vehicle control, significantly reduced the electric resistance of HCAEC monolayer between 2 and 10 h, consistent with increased permeability of the cardiac endothelial monolayer in response to the conditioned media (Fig. 6B, 6C).

### Conditioned media of miR-146a-5p–treated macrophages reduces CM contractility

**FIGURE 7.**
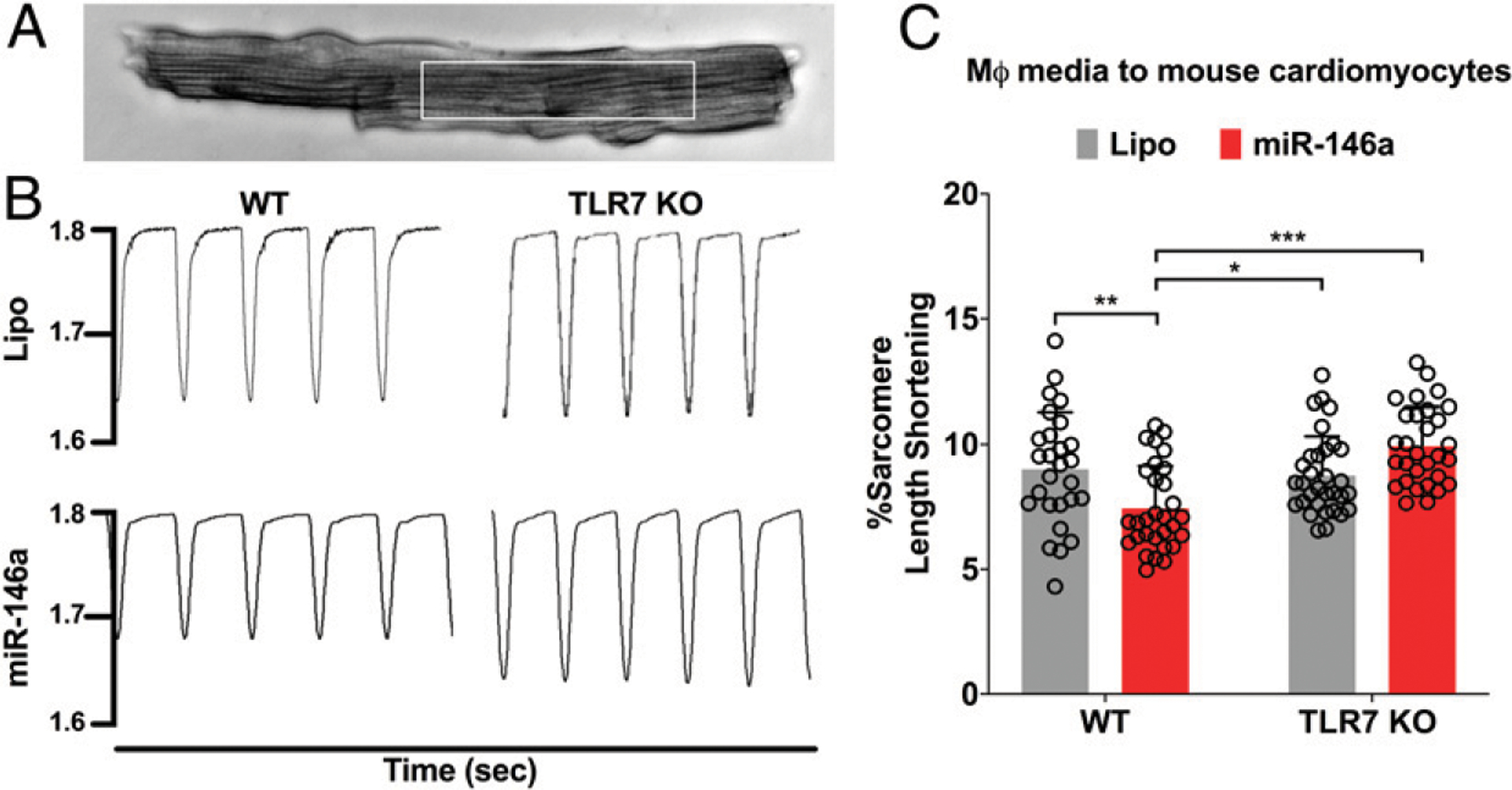
Macrophage-conditioned media reduce sarcomere shortening of adult mouse CMs. WT or TLR7 KO macrophages were treated for 16–18 h with either Lipo or miR-146a (50 nmol/l). Media were then collected and used at 50% concentration to treat WT adult mouse CMs for 2 h at 37°C. CMs were paced at 2 Hz and sarcomere length shortening was recorded using an IonOptix system. (**A**) A representative image of adult mouse CM. White box indicates sarcomere length measurement. (**B**) Representative tracings of sarcomere length shortening for WT and TLR7 KO CMs treated with Lipo or miR-146a-treated macrophage-conditioned media at 2 Hz. (**C**) Percentage of sarcomere length shortening. All treatments were blinded to the IonOptix operator (B.K.S.). *n* = 27–30 total CMs per group were analyzed from three separate CM preparations. Each error bar represents mean 6 SD. Data in (C) were analyzed with two-way ANOVA with Tukey post hoc test. *p < 0.05, **p < 0.01, ***p < 0.001. Lipo, lipofectamine; miR-146a, miR-146a-5p; MΦ, macrophage.

In addition to disrupting the endothelial barrier function, inflammation is known to compromise myocardial function and reduce CM contractility (32, 33). We tested whether miR-146a activation of TLR7 signaling in mouse bone marrow-derived macrophages, a cell type similar to myocardial macrophages derived from monocytes of blood circulation, could result in reduced CM sarcomere shortening through cross-talk between immune cells and CMs. We treated WT and TLR7 KO macrophages with miR-146a-5p for 16–18 h and harvested the conditioned media. We then isolated WT adult mouse CMs and treated them with 50% conditioned media from both WT and TLR7 KO macrophages for 2 h at 37°C. To avoid selection bias of the adult CMs for IonOptix recording as shown in Fig. 7A, all treatment groups were blinded to the operator (B.K.S.). CM sarcomere shortening was assessed using an IonOptix system (Fig. 7B). We discovered that the conditioned media from the WT macrophages treated with miR-146a-5p, as compared with that from vehicle-treated cells, significantly inhibited sarcomere shortening (9.00 ± 2.29 versus 7.43 ± 1.70%, 2 Hz, *n* = 27–30 per group from three mouse hearts, lipo versus miR-146a, *p* < 0.01) (Fig. 7C). Interestingly, the conditioned media collected from TLR7 KO cells, also treated with miR-146a-5p, failed to suppress the sarcomere shortening (Fig. 7C). Together, these data demonstrate that innate immune cells such as macrophages respond to miR-146a-5p treatment and consequently suppress CM sarcomere shortening.

## DISCUSSION

We and others have demonstrated that cellular nucleic acids such as RNA and miRNA are released from the heart following myocardial ischemic injury (4, 5, 7, 34–36). The cellular RNA, once released from the cells, can be highly proinflammatory by inducing cytokine production and innate immune cell activation (35). Systemic administration of RNase (4, 6) or a nucleic acid binding nanoprobe (5) reduces ex-RNA and attenuates myocardial cytokine production, innate immune cell infiltration, and cardiac injury following myocardial ischemia. These observations support the notion that ex-RNA may play a pivotal role in cardiac innate immune responses. Further analysis using miRNA array by our group identified a panel of plasma miRNAs that were upregulated following cardiac ischemia, some of which, such as miR-146a-5p, were highly proinflammatory in innate immune cells (7). But whether ex-miR-146a-5p is capable of inducing myocardial innate immune response and affecting cardiac function has been unclear. In this study, we demonstrated that miR-146a-5p loaded in engineered EVs induced a dose-dependent proinflammatory cytokine response in isolated CMs. A single dose of intracardiac injection of miR-146a-5p induced myocardial cytokine expression and robust monocyte/neutrophil recruitment into the heart. These innate immune responses appear to be mediated by TLR7 signaling. Moreover, miR-146a-5p induced HCAEC impairment by interrupting its barrier function. However, this effect appeared to be indirect and mediated through activated CMs and cardiac fibroblasts treated with miR-146a-5p. Finally, we demonstrated that miR-146a-5p was able to suppress CM sarcomere shortening through TLR7-mediated activation of myeloid-derived macrophages. All together, these data suggest that ex–miR-146a-5p may be an innate immune mediator capable of activating multiple cellular functions in the heart and modulate myocardial inflammation and CM function via TLR7 sensing.

Both adult mouse and neonatal rat CMs produced cytokines in response to miR-146a-5p treatment. The loss-of-function experiments in TLR7 KO CMs suggest that the cytokine response in adult CMs was clearly mediated through TLR7. Our initial pilot experiments using lipofectamine as the delivery vehicle of miRN As failed to show any cytokine response in adult CMs, likely due to poor transfection (23). However, miR-146a-5p loaded in EVs exhibited a strong proinflammatory effect in adult CMs. This is relevant because in vivo, circulating plasma miRNAs are carried in part by EVs. In fact, this has been implied in our previous work that demonstrated plasma EVs isolated from sepsis mice promoted significant CXCL2 and IL-6 via miRNA- and TLR7-dependent mechanisms (37). In addition to the physiological relevance of the EVs, engineered EVs could potentially be a means to deliver therapeutics to the heart following cardiac injury. Several studies have already demonstrated that delivery of EVs to the heart can lead to improved cardiac recovery after myocardial infarction and I/R injury (38, 39). Although these EV studies did not demonstrate that targeting miRNAs or other nucleic acids are potential treatments for cardiac injury, the study by Chen and colleagues used dextran-thiazole orange, a multivalent nucleic acid-scavenging nanoprobe, to illustrate ex localization of nucleic acids in the heart and, at the same time, to attenuate proinflammatory cytokine production and myocardial injury in a mouse model of I/R (5). Therefore, it is probable EVs containing molecules that target miRNAs and nucleic acids could be developed into therapies for cardiac injury.

We tested the impact of miR-146a-5p activation of CMs and cardiac fibroblasts on coronary artery endothelial barrier function. This is important because cardiovascular disease aggravates endothelial barrier dysfunction resulting in a significant increase in invading immune cells and myocardial inflammation (28, 29, 40). To examine miR-146a–mediated inflammatory signaling on endothelial function, we treated both CMs and cardiac fibroblasts with miR-146a and harvested the conditioned media to test endothelial permeability using an ECIS assay. We found that HCAEC monolayers responded with increased intercellular permeability to the conditioned media from both CMs and fibroblasts treated with miR-146a-5p. This indirect mechanism may be important because HCAECs do not express TLR7 and did not respond to miR-146a-5p directly (31). This data suggest that miR146a-5p–induced activation of CMs and fibroblasts may represent a potential mechanism that regulates cardiac endothelial barrier function. Although we speculate that both CMs and fibroblasts may secrete soluble mediators that cause endothelial dysfunction in response to ex-miRNAs, we do not know the nature of the mediators. Several cytokines have been reported to induce endothelial dysfunction, including TNF-α, IL-6, and IL-1b^42^. These cytokines exert their effects on endothelial cells through a variety of mechanisms such as increased expression of endothelial adhesion molecules to facilitate leukocyte transmigration or inhibition of NO synthase, both of which increase endothelial barrier permeability (41–43).

In addition to regulating endothelial barrier function, myocardial inflammation can contribute to cardiac dysfunction through complex inflammatory pathways. Proinflammatory cytokines initiate a localized inflammatory response (44), which triggers the expression of chemokines (45), cell adhesion molecules, and recruitment of leukocytes (46). Of the leukocytes, macrophages are of notable interest as inflammatory myeloid-derived macrophages produce large amounts of cytokinessuch as IL-6 and IL-18 through TLR signaling pathways (47, 48). Both IL-6 and IL-18 induce CM hypertrophy (47), and in both the noninfarcted and infarcted heart, IL-18 is also known to exacerbate fibrosis and cardiac dysfunction (48). In our study, we simulated interaction between macrophages and CMs and investigated whether activation of macrophagesby miR-146a → TLR7 signaling could affect adult CM contractility. We discovered that the conditioned media from WT, but not TLR7 KO, macrophages treated with miR-146a induced CM depression. These data suggest that TLR7 is necessary for miR-146a–induced cellular responses in macrophages and subsequent depression of CM contractility. We speculate that cytokines manufactured in macrophages following the miR-146a-5p treatment are likely the cause of CM depression as cytokines such as both IL-6 and TNF-α are known cardiac depressants (49, 50).

Our study suggests that endogenous ex–miR-146a could play a role in mediating cardiac cell cross-talk following cardiac injury. Although direct in vivo evidence is needed, we speculate that ex-miR-146a released from damaged tissue may be taken into endosomes in multiple cardiac cell types, including cardiac resident macrophages, fibroblasts, and CMs, activating the TLR7 signaling pathway and stimulating production of proinflammatory cytokines and chemokines such as IL-6 and CXCL-2. Cross-talk between these cells may then release additional cytokines and chemokines, resulting in disrupted endothelial barrier permeability or reduced contractility of the heart. This could, in turn, exacerbate the damage done by the initial tissue injury and lead to infiltration of innate immune cells that may contribute further to inflammatory responses following initial cardiac injury.

A number of limitations should be acknowledged. For one, this study was primarily in vitro. We did not establish the role of ex-miR-146a-5p in myocardial inflammation in vivo after transient ischemia. Another limitation was the use of intracardiac injections to deliver miR-146a to the heart, which was less physiologically relevant, but useful to test the myocardial response to exogenous miRNAs. Our goal was to test the concept that activation of miR-146a-5p → TLR7 signaling is sufficient to induce myocardial inflammation and CM contractile dysfunction. Loss-of-function and overexpression studies will help to define the exact role of miR-146a in myocardial inflammation and dysfunction after cardiac ischemic injury in vivo.

In summary, we identified that exogenous delivery of ss-miR-146a-5p was able to drive an innate immune response in the heart and on various cardiac cells, including CMs, coronary artery endothelial cells, and cardiac fibroblasts, thereby leading to myocardial inflammatory and cardiac cellular dysfunction. Heterocellular cross-talk between various cardiac cells leads to impairment of endothelial barrier function and CM depression following miR-146a → TLR7 activation. Overall, this study establishes a pivotal role of miR-146a → TLR7 signaling in myocardial innate immune response and CM dysfunction.

## Supplementary Material

1

## Abbreviations used in this article:

CM: cardiomyocyte
ECIS: electric cell substrate impedance–sensing
EV: ex vesicle
ex: extracellular
HCAEC: human coronary artery endothelial cell
I/R: ischemia-reperfusion
miRNA: microRNA
TLR7 KO: TLR7 knockout
VEGF: vascular endothelial growth factor
WT: wild-type

## References

[1] Yellon DM, and Hausenloy DJ. 2007 Myocardial reperfusion injury. N. Engl. J. Med 357: 1121–1135. 17855673 10.1056/NEJMra071667

[2] Wang GK, Zhu JQ, Zhang JT, Li Q, Li Y, He J, Qin YW, and Jing Q. 2010 Circulating microRNA: a novel potential biomarker for early diagnosis of acute myocardial infarction in humans. Eur. Heart J 31: 659–666. 20159880 10.1093/eurheartj/ehq013

[3] Creemers EE, Tijsen AJ, and Pinto YM. 2012 Circulating microRNAs: novel biomarkers and extracellular communicators in cardiovascular disease? Circ. Res 110: 483–495. 22302755 10.1161/CIRCRESAHA.111.247452

[4] Chen C, Feng Y, Zou L, Wang L, Chen HH, Cai JY, Xu JM, Sosnovik DE, and Chao W. 2014 Role of extracellular RNA and TLR3-Trif signaling in myocardial ischemia-reperfusion injury. J. Am. Heart Assoc 3: e000683. 24390148 10.1161/JAHA.113.000683 PMC3959703

[5] Chen HH, Yuan H, Cho H, Feng Y, Ngoy S, Kumar AT, Liao R, Chao W, Josephson L, and Sosnovik DE. 2017 Theranostic nucleic acid binding nanoprobe exerts anti-inflammatory and cytoprotective effects in ischemic injury. Theranostics 7: 814–825. 28382156 10.7150/thno.17366 PMC5381246

[6] Cabrera-Fuentes HA, Ruiz-Meana M, Simsekyilmaz S, Kostin S, Inserte J, Saffarzadeh M, Galuska SP, Vijayan V, Barba I, Barreto G, 2014 RNase1 prevents the damaging interplay between extracellular RNA and tumour necrosis factor-α in cardiac ischaemia/reperfusion injury. Thromb. Haemost 112: 1110–1119. 25354936 10.1160/TH14-08-0703

[7] Feng Y, Zou L, Yan D, Chen H, Xu G, Jian W, Cui P, and Chao W. 2017 Extracellular microRNAs induce potent innate immune responses via TLR7/MyD88-dependent mechanisms. J. Immunol 199: 2106–2117. 28768728 10.4049/jimmunol.1700730 PMC6462263

[8] Zou L, Feng Y, Xu G, Jian W, and Chao W. 2016 Splenic RNA and microRNA mimics promote complement factor B production and alternative pathway activation via innate immune signaling. J. Immunol 196: 2788–2798. 26889043 10.4049/jimmunol.1502106 PMC6464635

[9] Boldin MP, Taganov KD, Rao DS, Yang L, Zhao JL, Kalwani M, Garcia-Flores Y, Luong M, Devrekanli A, Xu J, 2011 miR-146a is a significant brake on autoimmunity, myeloproliferation, and cancer in mice. J. Exp. Med 208: 1189–1201. 21555486 10.1084/jem.20101823 PMC3173243

[10] Taganov KD, Boldin MP, Chang KJ, and Baltimore D. 2006 NF-kappaB-dependent induction of microRNA miR-146, an inhibitor targeted to signaling proteins of innate immune responses. Proc. Natl. Acad. Sci. USA 103: 12481–12486. 16885212 10.1073/pnas.0605298103 PMC1567904

[11] Akira S, Takeda K, and Kaisho T. 2001 Toll-like receptors: critical proteins linking innate and acquired immunity. Nat. Immunol 2: 675–680. 11477402 10.1038/90609

[12] Kawai T, and Akira S. 2010 The role of pattern-recognition receptors in innate immunity: update on toll-like receptors. Nat. Immunol 11: 373–384. 20404851 10.1038/ni.1863

[13] Roers A, Hiller B, and Hornung V. 2016 Recognition of endogenous nucleic acids by the innate immune system. Immunity 44: 739–754. 27096317 10.1016/j.immuni.2016.04.002

[14] Heil F, Hemmi H, Hochrein H, Ampenberger F, Kirschning C, Akira S, Lipford G, Wagner H, and Bauer S. 2004 Species-specific recognition of single-stranded RNA via toll-like receptor 7 and 8. Science 303: 1526–1529. 14976262 10.1126/science.1093620

[15] Diebold SS, Kaisho T, Hemmi H, Akira S, and Reis e Sousa C. 2004 Innate antiviral responses by means of TLR7-mediated recognition of single-stranded RNA. Science 303: 1529–1531. 14976261 10.1126/science.1093616

[16] Zhang Z, Ohto U, Shibata T, Krayukhina E, Taoka M, Yamauchi Y, Tanji H, Isobe T, Uchiyama S, Miyake K, and Shimizu T. 2016 Structural analysis reveals that toll-like receptor 7 is a dual receptor for guanosine and single-stranded RNA. Immunity 45: 737–748. 27742543 10.1016/j.immuni.2016.09.011

[17] Jian W, Gu L, Williams B, Feng Y, Chao W, and Zou L. 2019 Toll-like receptor 7 contributes to inflammation, organ injury, and mortality in murine sepsis. Anesthesiology 131: 105–118. 31045897 10.1097/ALN.0000000000002706 PMC6822621

[18] Jeyaram A, Lamichhane TN, Wang S, Zou L, Dahal E, Kronstadt SM, Levy D, Parajuli B, Knudsen DR, Chao W, and Jay SM. 2020 Enhanced loading of functional miRNA cargo via pH gradient modification of extracellular vesicles. Mol. Ther 28: 975–985. 31911034 10.1016/j.ymthe.2019.12.007 PMC7054713

[19] Lesizza P, Prosdocimo G, Martinelli V, Sinagra G, Zacchigna S, and Giacca M. 2017 Single-dose intracardiac injection of pro-regenerative microRNAs improves cardiac function after myocardial infarction. Circ. Res 120: 1298–1304. 28077443 10.1161/CIRCRESAHA.116.309589

[20] Lim CC, Apstein CS, Colucci WS, and Liao R. 2000 Impaired cell shortening and relengthening with increased pacing frequency are intrinsic to the senescent mouse cardiomyocyte. J. Mol. Cell. Cardiol 32: 2075–2082. 11040110 10.1006/jmcc.2000.1239

[21] Shi J, Guan J, Jiang B, Brenner DA, Del Monte F, Ward JE, Connors LH, Sawyer DB, Semigran MJ, Macgillivray TE, 2010 Amyloidogenic light chains induce cardiomyocyte contractile dysfunction and apoptosis via a non-canonical p38alpha MAPK pathway. Proc. Natl. Acad. Sci. USA 107: 4188–4193. 20150510 10.1073/pnas.0912263107 PMC2840082

[22] Matsui T, Li L, Wu JC, Cook SA, Nagoshi T, Picard MH, Liao R, and Rosenzweig A. 2002 Phenotypic spectrum caused by transgenic overexpression of activated Akt in the heart. J. Biol. Chem 277: 22896–22901. 11943770 10.1074/jbc.M200347200

[23] Louch WE, Sheehan KA, and Wolska BM. 2011 Methods in cardiomyocyte isolation, culture, and gene transfer. J. Mol. Cell. Cardiol 51: 288–298. 21723873 10.1016/j.yjmcc.2011.06.012 PMC3164875

[24] Hemmi H, Kaisho T, Takeuchi O, Sato S, Sanjo H, Hoshino K, Horiuchi T, Tomizawa H, Takeda K, and Akira S. 2002 Small antiviral compounds activate immune cells via the TLR7 MyD88-dependent signaling pathway. Nat. Immunol 3: 196–200. 11812998 10.1038/ni758

[25] Gao F, Kataoka M, Liu N, Liang T, Huang ZP, Gu F, Ding J, Liu J, Zhang F, Ma Q, 2019 Therapeutic role of miR-19a/19b in cardiac regeneration and protection from myocardial infarction. Nat. Commun 10: 1802. 30996254 10.1038/s41467-019-09530-1 PMC6470165

[26] Wagner JUG, and Dimmeler S. 2020 Cellular cross-talks in the diseased and aging heart. J. Mol. Cell. Cardiol 138: 136–146. 31783034 10.1016/j.yjmcc.2019.11.152

[27] Perbellini F, Watson SA, Bardi I, and Terracciano CM. 2018 Heterocellularity and cellular cross-talk in the cardiovascular system. Front. Cardiovasc. Med 5: 143. 30443550 10.3389/fcvm.2018.00143 PMC6221907

[28] Yang Q, He GW, Underwood MJ, and Yu CM. 2016 Cellular and molecular mechanisms of endothelial ischemia/reperfusion injury: perspectives and implications for postischemic myocardial protection. Am. J. Transl. Res 8: 765–777. 27158368 PMC4846925

[29] Prabhu SD, and Frangogiannis NG. 2016 The biological basis for cardiac repair after myocardial infarction: from inflammation to fibrosis. Circ. Res 119: 91–112. 27340270 10.1161/CIRCRESAHA.116.303577 PMC4922528

[30] Robilliard LD, Kho DT, Johnson RH, Anchan A, O’Carroll SJ, and Graham ES. 2018 The importance of multifrequency impedance sensing of endothelial barrier formation using ECIS technology for the generation of a strong and durable paracellular barrier. Biosensors (Basel) 8: 64. 10.3390/bios8030064 PMC6163417 29973526

[31] Opitz B, Hippenstiel S, Eitel J, and Suttorp N. 2007 Extra- and intracellular innate immune recognition in endothelial cells. Thromb. Haemost 98: 319–326. 17721613

[32] Nian M, Lee P, Khaper N, and Liu P. 2004 Inflammatory cytokines and postmyocardial infarction remodeling. Circ. Res 94: 1543–1553. 15217919 10.1161/01.RES.0000130526.20854.fa

[33] Boyd JH, Mathur S, Wang Y, Bateman RM, and Walley KR. 2006 Toll-like receptor stimulation in cardiomyoctes decreases contractility and initiates an NF-kappaB dependent inflammatory response. Cardiovasc. Res 72: 384–393. 17054926 10.1016/j.cardiores.2006.09.011

[34] Gidlöf O, Andersson P, van der Pals J, Götberg M, and Erlinge D. 2011 Cardiospecific microRNA plasma levels correlate with troponin and cardiac function in patients with ST elevation myocardial infarction, are selectively dependent on renal elimination, and can be detected in urine samples. Cardiology 118: 217–226. 21701171 10.1159/000328869

[35] Feng Y, Chen H, Cai J, Zou L, Yan D, Xu G, Li D, and Chao W. 2015 Cardiac RNA induces inflammatory responses in cardiomyocytes and immune cells via toll-like receptor 7 signaling. J. Biol. Chem 290: 26688–26698. 26363072 10.1074/jbc.M115.661835 PMC4646323

[36] Corsten MF, Dennert R, Jochems S, Kuznetsova T, Devaux Y, Hofstra L, Wagner DR, Staessen JA, Heymans S, and Schroen B. 2010 Circulating microRNA-208b and microRNA-499 reflect myocardial damage in cardiovascular disease. Circ. Cardiovasc. Genet 3: 499–506. 20921333 10.1161/CIRCGENETICS.110.957415

[37] Xu J, Feng Y, Jeyaram A, Jay SM, Zou L, and Chao W. 2018 Circulating plasma extracellular vesicles from septic mice induce inflammation via microRNA- and TLR7-dependent mechanisms. J. Immunol 201: 3392–3400. 30355788 10.4049/jimmunol.1801008 PMC6240609

[38] Gallet R, Dawkins J, Valle J, Simsolo E, de Couto G, Middleton R, Tseliou E, Luthringer D, Kreke M, Smith RR, 2017 Exosomes secreted by cardiosphere-derived cells reduce scarring, attenuate adverse remodelling, and improve function in acute and chronic porcine myocardial infarction. Eur. Heart J 38: 201–211. 28158410 10.1093/eurheartj/ehw240 PMC5837390

[39] Liu B, Lee BW, Nakanishi K, Villasante A, Williamson R, Metz J, Kim J, Kanai M, Bi L, Brown K, 2018 Cardiac recovery via extended cell-free delivery of extracellular vesicles secreted by cardiomyocytes derived from induced pluripotent stem cells. Nat. Biomed. Eng 2: 293–303. 30271672 10.1038/s41551-018-0229-7 PMC6159913

[40] Eltzschig HK, and Eckle T. 2011 Ischemia and reperfusion-from mechanism to translation. Nat. Med 17: 1391–1401. 22064429 10.1038/nm.2507 PMC3886192

[41] Kofler S, Nickel T, and Weis M. 2005 Role of cytokines in cardiovascular diseases: a focus on endothelial responses to inflammation. Clin. Sci. (Lond.) 108: 205–213. 15540988 10.1042/CS20040174

[42] Niebauer J, Dulak J, Chan JR, Tsao PS, and Cooke JP. 1999 Gene transfer of nitric oxide synthase: effects on endothelial biology. J. Am. Coll. Cardiol 34: 1201–1207. 10520813 10.1016/s0735-1097(99)00304-6

[43] True AL, Rahman A, and Malik AB. 2000 Activation of NF-kappaB induced by H(2)O(2) and TNF-alpha and its effects on ICAM-1 expression in endothelial cells. Am. J. Physiol. Lung Cell. Mol. Physiol 279: L302–L311. 10926553 10.1152/ajplung.2000.279.2.L302

[44] Brown MA, and Jones WK. 2004 NF-kappaB action in sepsis: the innate immune system and the heart. Front. Biosci 9: 1201–1217. 14977537 10.2741/1304

[45] Massey KD, Strieter RM, Kunkel SL, Danforth JM, and Standiford TJ. 1995 Cardiac myocytes release leukocyte-stimulating factors. Am. J. Physiol 269: H980–H987. 7573543 10.1152/ajpheart.1995.269.3.H980

[46] Simms MG, and Walley KR. 1999 Activated macrophages decrease rat cardiac myocyte contractility: importance of ICAM-1-dependent adhesion. Am. J. Physiol 277: H253–H260. 10409204 10.1152/ajpheart.1999.277.1.H253

[47] Kobara M, Noda K, Kitamura M, Okamoto A, Shiraishi T, Toba H, Matsubara H, and Nakata T. 2010 Antibody against interleukin-6 receptor attenuates left ventricular remodelling after myocardial infarction in mice. Cardiovasc. Res 87: 424–430. 20211866 10.1093/cvr/cvq078

[48] Toldo S, Mezzaroma E, O’Brien L, Marchetti C, Seropian IM, Voelkel NF, Van Tassell BW, Dinarello CA, and Abbate A. 2014 Interleukin-18 mediates interleukin-1-induced cardiac dysfunction. Am. J. Physiol. Heart Circ. Physiol 306: H1025–H1031. 24531812 10.1152/ajpheart.00795.2013 PMC3962640

[49] Yu X, Kennedy RH, and Liu SJ. 2003 JAK2/STAT3, not ERK1/2, mediates interleukin-6-induced activation of inducible nitric-oxide synthase and decrease in contractility of adult ventricular myocytes. J. Biol. Chem 278: 16304–16309. 12595539 10.1074/jbc.M212321200

[50] Saini HK, Xu YJ, Zhang M, Liu PP, Kirshenbaum LA, and Dhalla NS. 2005 Role of tumour necrosis factor-alpha and other cytokines in ischemia-reperfusion-induced injury in the heart. Exp. Clin. Cardiol 10: 213–222. 19641672 PMC2716235

