# Supplementary material for: Extracellular miR-146a-5p Induces Cardiac Innate Immune Response and Cardiomyocyte Dysfunction
Source: Immunohorizons. Author manuscript; Available in PMC 2020 Dec 22. (PMC7754174; doi:10.4049/immunohorizons.2000075)
Supplement: 1 [file NIHMS1652950-supplement-1.pdf]

Supplemental Figures

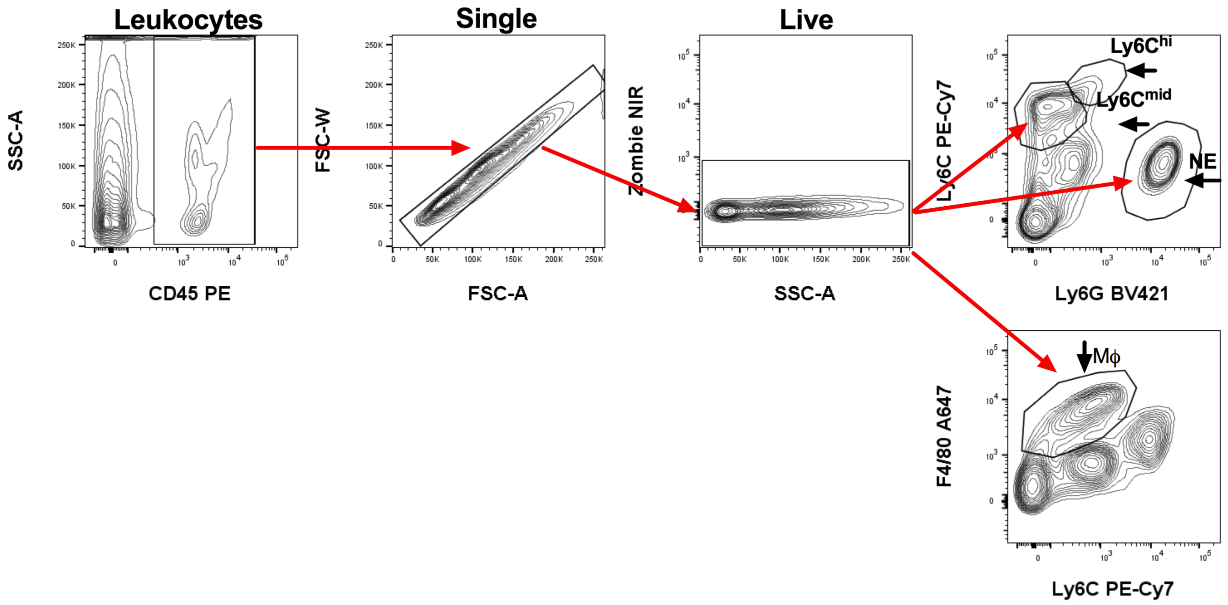

Supplemental Figure 1. Gating strategy for flow cytometry analysis. CD45+ leukocytes were gated first and followed by singlets and live cells. Ly6C^{high} and Ly6C^{mid} and Ly6G^{neg} cells were gated as monocytes. Ly6G^{pos} cells were gated as neutrophils. F4/80^{+} and Ly6C^{neg} cells were gated as macrophages. NE, neutrophils; Mϕ, macrophages.

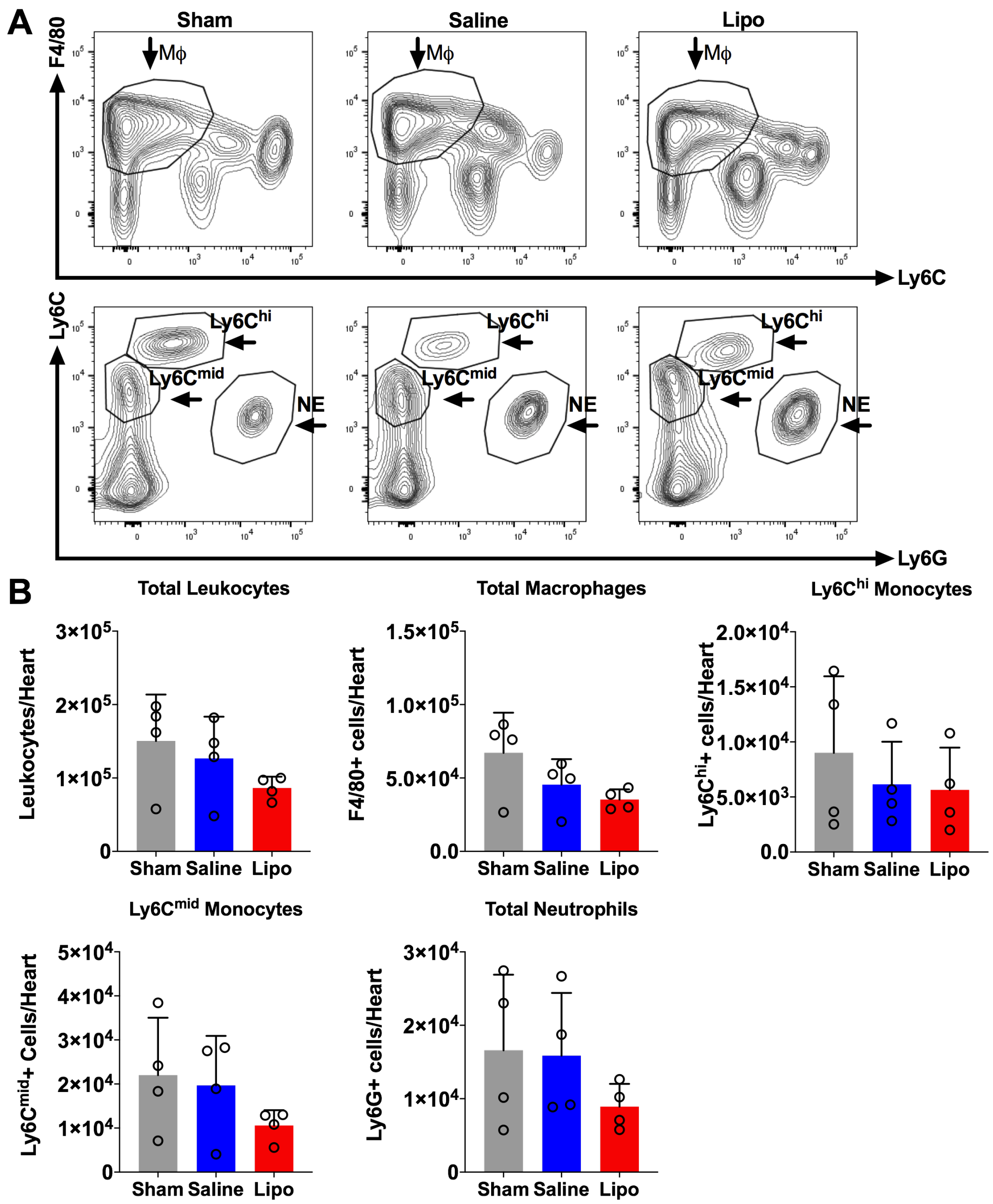

Supplemental Figure 2. Intracardiac injection of saline or lipofectamine does not elicit any innate immune cell infiltration as compared with Sham without any injection. After thoracotomy, hearts were injected with 60 µL saline or 10 µL of lipofectamine mixed with 50 µL of saline or were given no injection (sham). **A.** Representative flow contour plot for each group. **B.** The total leukocytes, macrophages, monocytes and neutrophils for each group. Each error bar represents mean ± SD, $n = 4$ mice per group, P = NS. Data in all panels was analyzed with one-way ANOVA with Tukey's post hoc test. Lipo, lipofectamine; Mϕ, macrophage; NE, neutrophil.
